# Combined cellular and biochemical profiling of Bruton’s tyrosine kinase inhibitor nemtabrutinib reveals potential application in MAPK-driven cancers

**DOI:** 10.3389/fonc.2025.1667291

**Published:** 2025-10-22

**Authors:** Daphne J. F. Kluitmans, Janneke J. T. M. Melis, Esmee van den Bossche, Jacob Ytsma, Jeroen A. D. M. de Roos, Oscar P. J. van Linden, Yvonne Grobben, Jeffrey J. Kooijman, Guido J. R. Zaman

**Affiliations:** ^1^ Oncolines B.V., Oss, Netherlands; ^2^ Division of Innovations in Human Health and Life Sciences, Amsterdam Institute of Molecular and Life Sciences, Faculty of Science, Vrije Universiteit Amsterdam, Amsterdam, Netherlands

**Keywords:** cancer cell line proliferation, Bruton’s tyrosine kinase (BTK), bioinformatics, kinase profiling, MAPK, Biacore, kinase inhibitor

## Abstract

**Background:**

Nemtabrutinib is a reversible inhibitor of both wild-type and acquired resistance-related mutant BTK. Since nemtabrutinib biochemically inhibits various kinases, new drug response biomarkers, cross-reactivities and differentiators may be identified.

**Methods:**

Nemtabrutinib was profiled in a large panel of cancer cell line viability assays. The sensitivity profile of nemtabrutinib was compared with the profiles of 135 kinase inhibitors across the same cell lines. Additionally, cell line sensitivity was related to gene mutation status, gene and protein expression levels, and gene dependency scores. Potential targets were explored using biochemical assays.

**Results:**

Sensitivity to nemtabrutinib is on average three times higher in *BRAF-*mutant *versus* wild-type cell lines. Consistently, the sensitivity profile of nemtabrutinib is similar to that of MEK, ERK and pan-RAF inhibitors. Furthermore, sensitivity to nemtabrutinib is correlated with high *FGFR3* gene expression levels, high levels of phosphorylated MEK1 and genetic dependency on several mitogen-activated protein kinases (MAPK). Biochemical profiling confirms that nemtabrutinib inhibits several growth factor receptor tyrosine kinases and downregulates MAPK signaling via MEK. Molecular docking studies suggest that nemtabrutinib preferentially binds in the ATP-binding pocket of MEK1.

**Conclusion:**

Combined cancer cell panel and biochemical profiling reveals previously underappreciated cross-reactivities of nemtabrutinib indicating a potential application in MAPK-driven cancers.

## 1 Background

Cancer cell panel profiling is the parallel testing of drugs on large panels of human cancer cell lines in cell viability assays (1). Predictive biomarkers of drug response can be identified by relating drug sensitivity to genomic information of the cell lines (2–7). In addition, comparing the sensitivity profile of different drugs profiled on the same cell line panel can help in elucidating their biochemical mechanisms of action (8). For example, the profile of the Bruton’s tyrosine kinase (BTK) inhibitor ibrutinib in the Oncolines^®^ cancer cell line panel shows similarity with that of epidermal growth factor receptor (EGFR) tyrosine kinase inhibitors (5). This is in accordance with the biochemical inhibition of EGFR by ibrutinib (9), which has been related to its major clinical adverse effects (diarrhea and rash) (10).

BTK plays a key role in oncogenic B-cell signaling and is a molecular target for the development of therapies against various B-cell malignancies. Currently, four small-molecule BTK inhibitors have been approved by the U.S. Food and Drug Administration (FDA) for treatment of mantle cell lymphoma, chronic lymphocytic leukemia and small lymphocytic lymphoma. The first three approved drugs (*i.e.*, ibrutinib, acalabrutinib and zanubrutinib) are covalent inhibitors that bind irreversibly to a cysteine residue (C481) in the active site of BTK. In more than 50% of patients treated with these inhibitors, clinical drug resistance is associated with amino acid substitutions at position C481 in BTK (11–13). These substitutions, which mostly involve conversion to serine, but also to arginine, preclude the binding of covalent inhibitors (11–13). To overcome this mechanism of acquired resistance, the reversible inhibitors pirtobrutinib (LOXO-305) (14) and nemtabrutinib (MK-1026; ARQ 531) (15) targeting both wild-type and C481-mutant BTK were developed. Pirtobrutinib received market authorization in 2023, while nemtabrutinib is still under investigation in phase 3 clinical trials for B-cell malignancies (16).

In this study, cell panel profiling experiments and bioinformatic analyses were performed for nemtabrutinib to identify predictive drug response biomarkers and differentiators towards approved BTK inhibitors. Several kinases not previously described to be involved in the cellular response of nemtabrutinib, including various mitogen-activated protein kinases (MAPKs), were identified as potential predictive drug response markers. Biochemical kinase assays confirmed that some of these kinases are a direct molecular target of nemtabrutinib.

## 2 Methods

### 2.1 BTK inhibitors

Nemtabrutinib and pirtobrutinib were purchased from ChemScene LLC (Monmouth Junction, NJ, USA). Acalabrutinib, ibrutinib and zanubrutinib were purchased from MedChemExpress LLC (Monmouth Junction, NJ, USA), Axon Medchem B.V. (Groningen, the Netherlands), and Activate Scientific GmbH (Prien am Chiemsee, Germany), respectively. All inhibitors were stored as dry powders at 4 °C and were dissolved in dimethyl sulfoxide (DMSO) at 10 mmol/L concentration before testing.

### 2.2 Kinase assays

Nemtabrutinib and the approved BTK inhibitors were profiled on a panel of 254 wild-type kinases in mobility shift assays (MSA) at Carna Biosciences, Inc. (Kobe, Japan). Kinase inhibition was measured at a compound concentration of 1 µmol/L and an ATP concentration of *K*_M,bin_ (17), which is an ATP concentration within 2-fold of the affinity of an individual kinase for ATP (*K*_M,ATP_). Percentage inhibition values were mapped to a phylogenetic kinome tree using Coral (http://phanstiel-lab.med.unc.edu/CORAL/, accessed September 4^th^, 2024) (18). Half-maximal inhibitory concentration (IC_50_) values were determined using duplicate 10-point dilution series in MSA at Carna Biosciences, Inc. for 15 kinases, while for MEK1 and MEK2, inhibition of enzymatic activity was measured in-house in enzyme-linked immunosorbent assays (ELISA) (Carna Biosciences, Inc., cat. no. 07–41 and 07-42). Percentage inhibition of MEK1 and MEK2 at 1 µmol/L nemtabrutinib and *K*_M,bin_ ATP concentration was derived from the dose-response curve as well and mapped to the phylogenetic kinome tree, as described above. Inhibition of MLK1 and B-RAF at *K*_M,ATP_ was determined in a radiometric assay using ^32^P-labeled ATP at Eurofins Cerep SA (Celle-Lévescault, France). Interaction with SIK3 was assessed in a competition binding assay at Eurofins DiscoverX LLC (San Diego, CA, USA). The binding of nemtabrutinib to biotinylated, inactive MEK1 (Carna Biosciences, Inc., cat. no. 07-441-10-20N) and activated B-RAF (Carna Biosciences, Inc., cat. no. 09-422-20N) was measured by surface plasmon resonance (SPR) using a Biacore 1S+ (Cytiva), as described previously (9).

### 2.3 Cell line panel

A panel of 160 cancer cell lines was used in this study. **Supplementary Table S1** provides an overview of the 160 cell lines used for cell viability assays, the subset of 102 cell lines used for comparative profiling and the subsets used for different bioinformatic analyses, which were dependent on the availability of public data. Cell lines were purchased from the American Type Culture Collection (ATCC) (Manassas, VA, USA), the German Collection of Microorganisms and Cell Cultures (DSMZ) (Braunschweig, Germany), the RIKEN BioResource Research Center (Tsukuba, Ibaraki, Japan) or the Japanese Collection of Research Bioresources (JCRB) (Ibaraki City, Osaka, Japan). All cell lines were propagated in the cell culture media as indicated in **Supplementary Table S1**. Cell viability assays were carried out within ten passages of the original vials. The authenticity of the ATCC and DSMZ cell lines has been confirmed by short tandem repeat analysis at both institutions. In addition, the mutation status of several cancer genes has been confirmed in various cell lines, including RIKEN and JCRB cell lines, by next-generation sequencing of genomic DNA isolated from the same cell batches as used for the viability assays (19, 20).

### 2.4 Cell viability assays

Intracellular ATP content was used as an indirect readout of cell number using the ATPlite 1Step bioluminescence assay (Revvity, Groningen, the Netherlands). Cells were seeded in 384-well plates at an optimized density to ensure unrestricted growth and maximal signal at the end of the experiment. After 24 hours of incubation, the starting cell number was determined by adding ATPlite to each well of a control plate and recording luminescence on an Envision multimode reader (Revvity, Waltham, MA, USA) (4). Compounds were diluted in DMSO in √10-fold steps from 10 mmol/L stocks to obtain 9-point dilution series. After further 31.6-fold dilution in 20 mmol/L HEPES (pH 7.4), the dilution series were added in duplicate to the cells in the 384-well plates to determine the effect of the compounds on cell viability. Vehicle-treated controls were included to determine maximal cell proliferation. The final DMSO concentration was 0.4% (v/v) in all wells. After incubation for an additional 72 hours, the ATP content was measured in each well using ATPlite. The luminescent signal in inhibitor-treated wells was normalized to vehicle-treated control wells to determine the percentage viability at each concentration. Cell doublings were determined by relating the cell number of vehicle-treated controls to the starting cell number for each cell line. The viability assay of a cell line was repeated when the cell doubling deviated > 2-fold from the historic doubling as determined by multiple independent experiments. The quality of the complete assay was determined by a parallel test with doxorubicin on two cell lines. IC_50_ values were calculated by fitting a four-parameter logistic model to the percentage viability values using IDBS XLfit5 (IDBS, Guildford, United Kingdom). All curves were visually inspected and submitted to an F-test as implemented in IDBS XLfit5. Curves with an F-value above 1.5 were invalidated and IC_50_ values were maximized at the highest concentration. The highest initial test concentration in each dose range was 31.6 µmol/L and the lowest was 3.16 nmol/L. When this dose range was too high in a certain cell line to allow for a reliable determination of the IC_50_, a new dilution series was prepared using a diluted stock solution and was used to retest the compound on the cell line.

### 2.5 Datasets

Multiple datasets were used for the bioinformatic analyses. The gene mutation, fusion and amplification status of the cell lines were retrieved from the COSMIC Cell Lines Project available at the COSMIC database (https://cancer.sanger.ac.uk) (21), the DepMap database (release 24Q2) (7) and literature. Mutations were filtered for oncogenic relevance with the Cancer Hotspots database (https://www.cancerhotspots.org/#/home) (22) and the OncoKB database (https://www.oncokb.org/) (23, 24), as described previously (6). Gene mutation data are available for 156 of the 160 profiled cell lines (**Supplementary Table S1**).

The in-house Oncolines^®^ kinase inhibitor dataset contains the IC_50_ profiles of 135 kinase inhibitors that have been profiled on a subset of 66 or 102 of the 160 cancer cell lines. The kinase inhibitor set includes approved drugs, clinical and pre-clinical inhibitors, and tool compounds (**Supplementary Table S2**).

Gene and protein expression data of cell lines as well as gene dependency scores were retrieved from the DepMap database (7). Basal mRNA gene expression levels (release 23Q4) were used and are available for 145 of the 160 profiled cell lines (**Supplementary Table S1**). Protein expression levels, as determined by Reverse Phase Protein Array (release 22Q2), were used and are available for 128 of the 160 cancer cell lines (**Supplementary Table S1**).

Gene dependency scores from a large individual CRISPR knock-out screen and large RNA interference (RNAi) knock-down screens (release 23Q4 and Demeter Data v6, respectively) were used (25–29). In these screens, the effect of single-gene knock-out or knock-down on cell viability has been determined. The more negative a dependency score, the more dependent a cell line is on a specific gene. The CRISPR dataset includes data for 120 of the 160 profiled cell lines, while the RNAi dataset covers data for 107 of the 160 cell lines (**Supplementary Table S1**).

### 2.6 Bioinformatics

For all bioinformatic analyses of cell viability assay data, ^10^logIC_50_ values (in nmol/L) were used as a measure for cell line sensitivity. All calculations were performed in R (version 4.3.1) (30).

For the gene mutation analysis, profiled cell lines were classified as having an alteration in a gene if the gene was mutated, fused, amplified (oncogenes) or deleted (tumor suppressor genes). Otherwise, cell lines were classified as ‘wild-type’. The analysis focused on a subset including known oncogenic kinase genes (31) of which only 23 genes that were altered in three or more cell lines were included to allow for proper statistics. A type II analysis of variance (ANOVA) was performed to identify significant associations between cell line sensitivity to nemtabrutinib and kinase gene alterations. Significance after multiple testing correction was determined with the Benjamini-Hochberg procedure (*i.e.*, false discovery rate < 20%).

The IC_50_ profile of nemtabrutinib was compared to the IC_50_ profiles of the kinase inhibitors in the Oncolines^®^ kinase inhibitor dataset by calculating the Pearson correlation between compounds or by hierarchical clustering. The network tree of inhibitors and Pearson correlations was generated using the Fruchterman–Reingold algorithm, as implemented in the R package igraph (32). Hierarchical clustering of cell panel viability data from the kinase inhibitors was performed with the Ward method, using 1 – Pearson correlation (*r*) as clustering distance, as described previously (8).

The IC_50_ values of nemtabrutinib were correlated to basal gene expression levels of 371 genes and protein expression levels of 55 genes that have been experimentally determined to be involved in cancer (33) using Pearson correlations. Additionally, Pearson correlations were determined between the IC_50_ values of nemtabrutinib and CRISPR or RNAi dependency scores of 465 and 204 kinase genes, respectively (34). Significance of all correlations was determined by calculating *p-*values, which were subjected to the Benjamini-Hochberg procedure to correct for multiple testing. Correlations with a false discovery rate of < 20% were considered significant.

### 2.7 Computational modeling

Docking of nemtabrutinib in the ATP- and allosteric binding pocket of MEK1 was performed with the structure of MEK1 with the ATP-competitive MEK1 inhibitor DS03090629 (PDB ID:7XLP (35)) and the structure of MEK1 with adenosine 5’-(β,γ-methylene)triphosphate (AMP-PCP) and allosteric MEK1 inhibitor cobimetinib (PDB ID: 4AN2 (36)), respectively. Prior to docking, the structures were processed in MOE v2024.06, hydrogen atoms were added, partial charges were calculated using the Amber10:EHT forcefield and energy minimization was performed. The ATP- and allosteric binding pockets were selected as amino acids within 4.5 Å distance of DS03090629 and cobimetinib, respectively. DS03090629, cobimetinib and co-factors not involved in ligand binding were removed from the structures. Docking in the allosteric binding pocket was done in the presence of AMP-PCP and magnesium.

Docking was performed using the Amber10:EHT forcefield with Triangle Matcher placement and Induced Fit receptor refinement. Redocking of DS03090629 and cobimetinib in their respective protein structures validated the docking procedure (RMSDs: 0.61 and 0.92; docking scores: -12.2 and -10.1). Docking scores for nemtabrutinib in the ATP-binding pocket ranged from -10.9 to -10.5. For docking of nemtabrutinib in the allosteric binding pocket, 100 docking poses were generated of which the 25 highest-scoring poses after refinement (-9.8 to -8.9) were reported. Docking poses were visually inspected and analyzed for ligand-protein interactions using interaction fingerprints (IFPs).

## 3 Results

### 3.1 Kinome profiling

**Figure 1:**
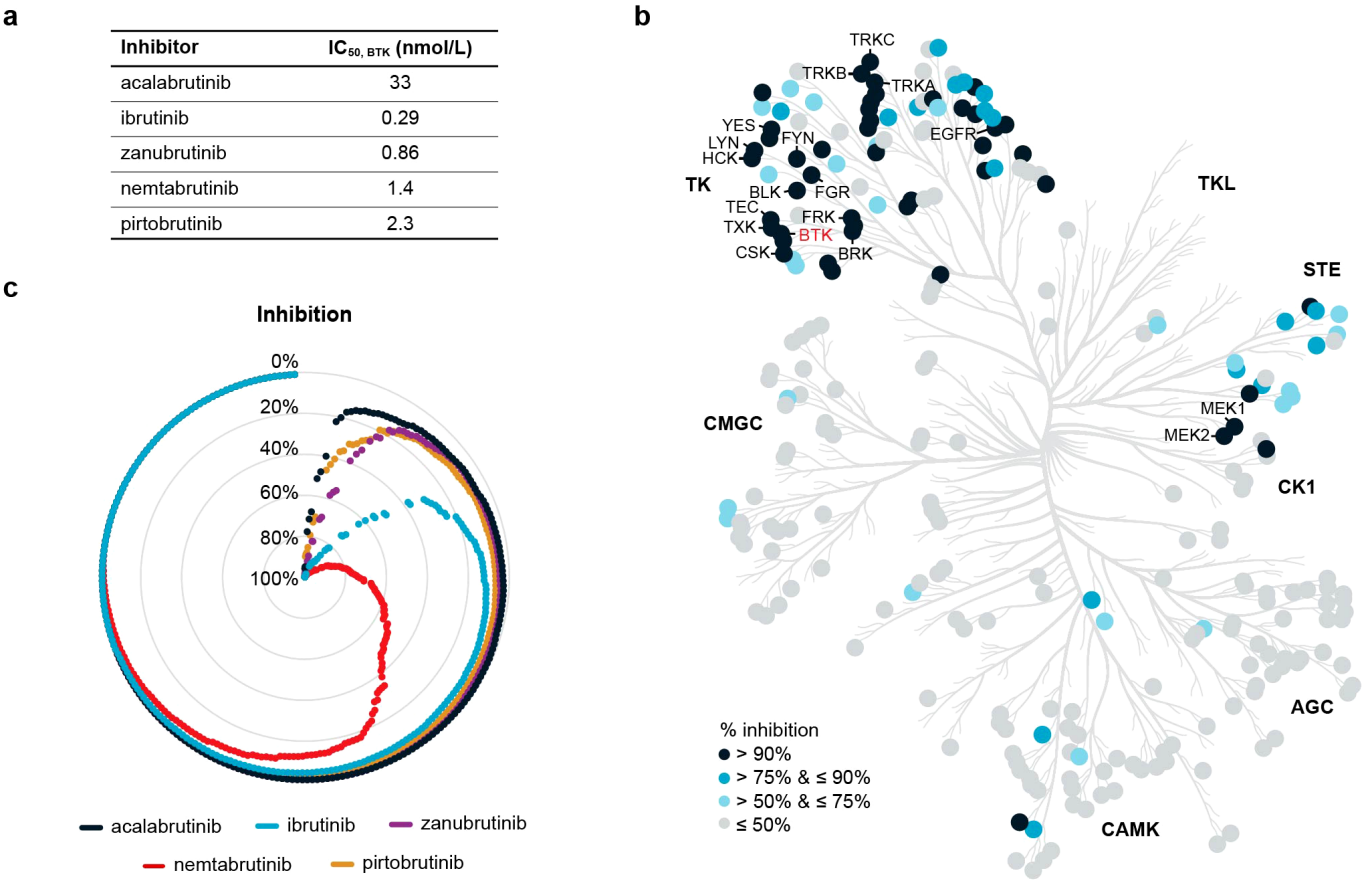
Biochemical kinase profiling. **(a)** Biochemical IC_50_ values of nemtabrutinib and four approved BTK inhibitors on BTK. **(b)** Phylogenetic tree of human protein kinases highlighting 256 wild-type kinases that were examined for inhibition by nemtabrutinib in profiling experiments. Enzyme assays were performed at *K*_M,bin_ ATP and 1 µmol/L nemtabrutinib. **(c)** Radar chart of the percentage inhibition of 254 wild-type kinases in the presence of 1 µmol/L nemtabrutinib or one of the four approved BTK inhibitors. Each dot represents a kinase and the kinases are ordered based on percentage inhibition per inhibitor.

Nemtabrutinib inhibited the tyrosine kinase activity of wild-type BTK in biochemical assays with an IC_50_ of 1.4 nmol/L, which is in range with the inhibition of BTK by the approved BTK inhibitors (**Figure 1a**). The acquired resistance-related C481S-mutant BTK is inhibited by nemtabrutinib with a similar IC_50_ (1.6 nmol/L, **Table 1**). As previously noted (15), nemtabrutinib shows cross-reactivity with many other kinases in biochemical assays, including other TEC family, SRC family and growth factor receptor tyrosine kinases (RTKs) (**Figure 1b**, **Table 1**). When profiled on a panel of 254 wild-type kinases at a single concentration of 1 µmol/L, nemtabrutinib inhibited the activity of 63 kinases by more than 75% and 45 kinases by more than 90% (**Figure 1b**, **Supplementary Table S3**). This is a considerably higher number of cross-reactivities than found for the approved BTK inhibitors on the same kinase panel (**Figure 1c**, **Supplementary Table S3**). For example, the other reversible BTK inhibitor pirtobrutinib inhibited only five kinases by more than 75% (*i.e.*, Aurora B, BRK, CSK, EGFR and TEC) (**Supplementary Table S3**). These results indicate that nemtabrutinib has a broader selectivity in biochemical assays compared to pirtobrutinib and the approved irreversible BTK inhibitors.

**Table 1:** IC_50_ of nemtabrutinib in kinase enzyme activity assays performed at *K*_M__,__bin_ ATP or 1 mmol/L ATP.

Kinase		IC_50_ in nmol/L	
Gene	Protein	*K*_M,bin_ ATP	1 mmol/L ATP
*BRAF*	B-RAF	73.0^1^	335
*BRAF* [V600E]	B-RAF [V600E]		220
*BTK*	BTK	1.42	
*BTK* [C481S]	BTK [C481S]	1.60	
*FGFR1*	FGFR1	134	
*FGFR2*	FGFR2	34.0	
*FGFR3*	FGFR3	70.1	
*FGFR4*	FGFR4	1,400	
*MAP2K1*	MEK1	8.46	456
*MAP2K2*	MEK2	9.50	494
*MAP3K9*	MLK1^1^	4,870	
*MAPK3*	ERK1	543	
*MAPK1*	ERK2	505	
*PDGFRA*	PDGFRα	12.4	
*SIK3*	SIK3	> 10,000^1,2^	9,320
*TEC*	TEC	0.750	
*YES*	YES	0.591	

^1^Assays performed at Eurofins. All other assays were performed at Carna biosciences, Inc. or in-house.

^2^Assay was a ligand competition assay instead of an enzyme activity assay.

### 3.2 Cancer cell line profiling

**Figure 2:**
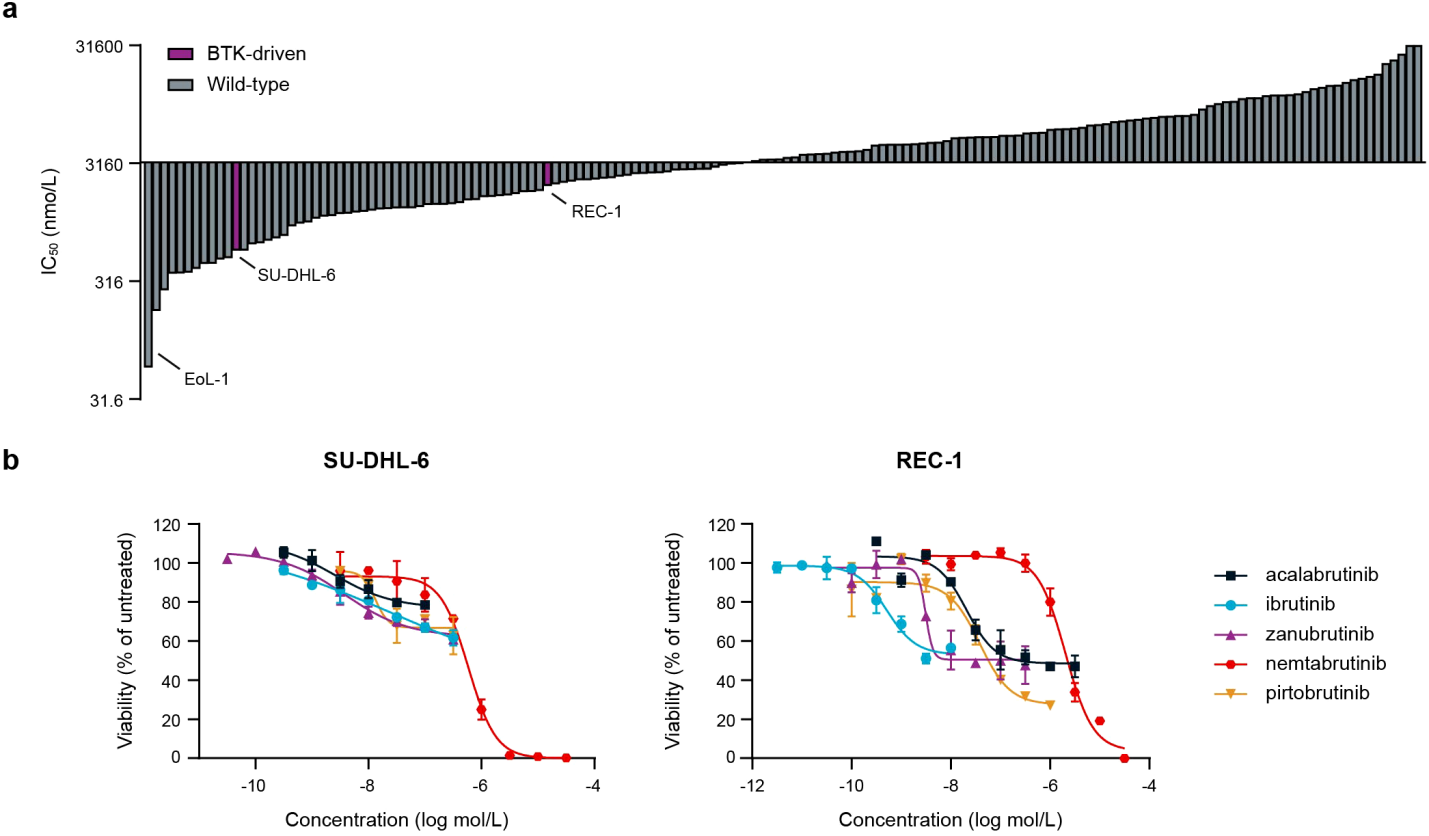
Cancer cell panel profiling of nemtabrutinib. **(a)** Waterfall plot of IC_50_ values of nemtabrutinib in cell viability assays with 160 cancer cell lines. **(b)** Dose-response curves of nemtabrutinib and the four approved BTK inhibitors in viability assays with SU-DHL-6 and REC-1 cells.

To study the activity and selectivity of nemtabrutinib in cancer cells, nemtabrutinib was profiled on a panel of 160 human cancer cell lines in cell viability assays. The cell panel represents a wide range of solid tumors and hematological malignancies (**Supplementary Table S1**). Nemtabrutinib demonstrated potent inhibitory activity across cell lines from all tissues and disease types. The most sensitive cell line was the chronic eosinophilic leukemia cell line EoL-1 (IC_50_ = 59 nmol/L), expressing a fusion gene of Factor Interacting with PAPOLA and CPSF1 (FIP1L1) and platelet-derived growth factor receptor α (PDGFRα) (**Figure 2a**, **Supplementary Table S4**) (37). Other cell lines among the most sensitive responders were two T-cell acute lymphoblastic leukemia (T-ALL) cell lines (Jurkat E6.1 and CCRF-HSB-2) and several cell lines derived from solid tumors, including endometrial (HEC-251 and HEC-1-B) and colon cancers (HT-29). The germinal center B-cell like diffuse large B-cell lymphoma (DLBCL) cell line HT and the prostate carcinoma cell line LNCaP clone FGC were among the least sensitive cell lines in the viability assays (IC_50_ > 31.6 µmol/L) (**Supplementary Table S4).**

The profiled cancer cell line panel contains two cell lines derived from BTK-dependent cancers, *i.e.*, the DLBCL cell line SU-DHL-6 and the mantle cell lymphoma cell line REC-1. The SU-DHL-6 cell line harbors a mutation in *MYD88* resulting in the activation of BTK (38), while the REC-1 cell line has constitutively active B-cell receptor signaling (39). Nemtabrutinib inhibited the viability of these cell lines with an IC_50_ of 0.6 µmol/L and 2.0 µmol/L, respectively. This contrasts the potent IC_50_ values of the irreversible BTK inhibitors ibrutinib, acalabrutinib and zanubrutinib on these cell lines (**Figure 2b**, **Supplementary Table S5**). However, the approved BTK inhibitors showed only partial effects, while nemtabrutinib reached almost complete inhibition of viability (**Figure 2b**).

### 3.3 Bioinformatic analysis of cell panel profiling data

To investigate which kinases could be responsible for the broad cellular activity of nemtabrutinib, a number of bioinformatic analyses were performed on the cell panel profiling data. Both genomic alterations and aberrant expression of genes or proteins can induce tumor cell proliferation (31, 33). Therefore, we correlated the IC_50_ profile of nemtabrutinib with the mutation status of oncogenic kinases, the gene dependency scores of human kinase genes and with the gene or protein expression levels of known cancer genes in the cell lines.

**Figure 3:**
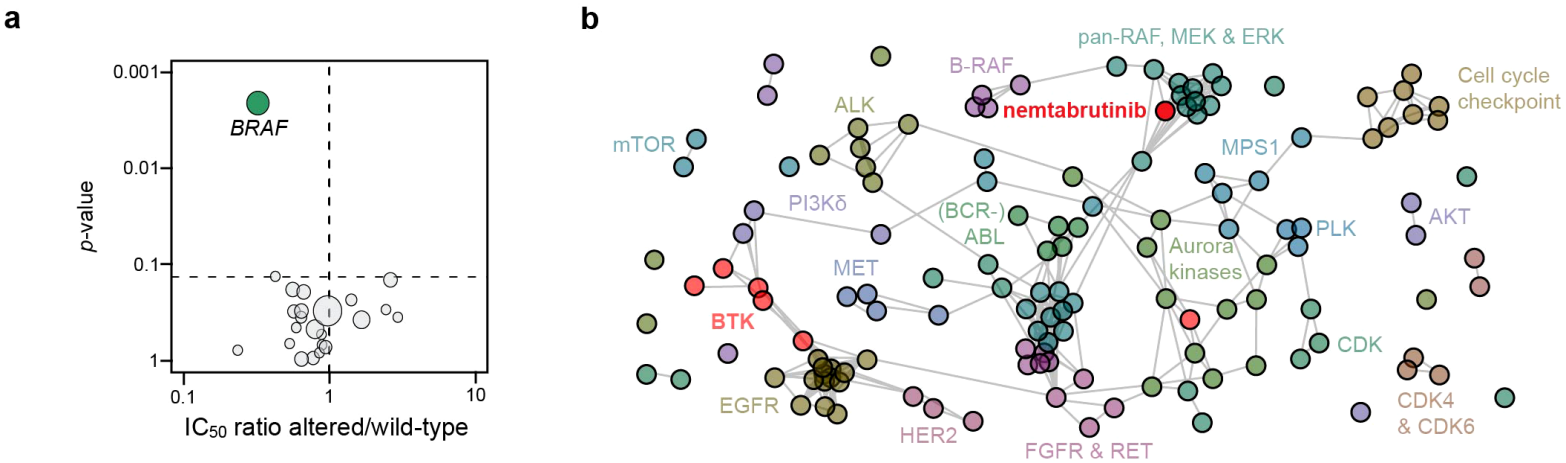
Cancer gene mutation analysis and comparative profiling. **(a)** Volcano plot showing the correlation of nemtabrutinib response in cell viability assays with the mutation status of 23 oncogenic kinases in the cell lines. Each circle represents a kinase gene that is mutated in at least three cell lines. **(b)** Network tree connecting inhibitors with a similar profile. In case the Pearson correlation coefficient of the IC_50_ fingerprint of two compounds is 0.5 or higher, a connection is drawn in the network. The length of the line has no meaning. Nemtabrutinib and the BTK inhibitors are colored in red.

Associations between cell line sensitivity and gene mutations were determined by ANOVA. Of the 23 kinase genes analyzed, only the mutation status of *BRAF* was found to be significantly associated with nemtabrutinib sensitivity (**Figure 3a**). Cancer cell lines harboring mutations in *BRAF* were on average three times more sensitive than cell lines expressing wild-type *BRAF.* Mutant *BRAF* is also a predictive biomarker of drug response for B-RAF and MEK1 inhibitors profiled in our cancer cell line panel (4, 6).

To further investigate the mechanism underlying the association of cell line sensitivity to nemtabrutinib with *BRAF* mutation status, we compared the IC_50_ profile of nemtabrutinib with the profiles of the 135 kinase inhibitors in the Oncolines^®^ kinase inhibitor dataset in a one-to-one comparison analysis. Inhibitors are considered to have a similar inhibitory profile if the Pearson correlation is 0.5 or higher. If inhibitors show a similar profile, they are connected to each other in a network tree for visualization (**Figure 3b**). The analysis revealed that nemtabrutinib is not connected with the approved BTK inhibitors, but shows connections with MEK, ERK and pan-RAF inhibitors (**Figure 3b**). A comparative analysis based on an unsupervised hierarchical clustering showed similar results (**Supplementary Figure S1**). The approved BTK inhibitors cluster at distant locations in the hierarchical clustering tree. For instance, and as noted before (8), ibrutinib clusters with EGFR inhibitors (**Supplementary Figure S1**).

**Figure 4:**
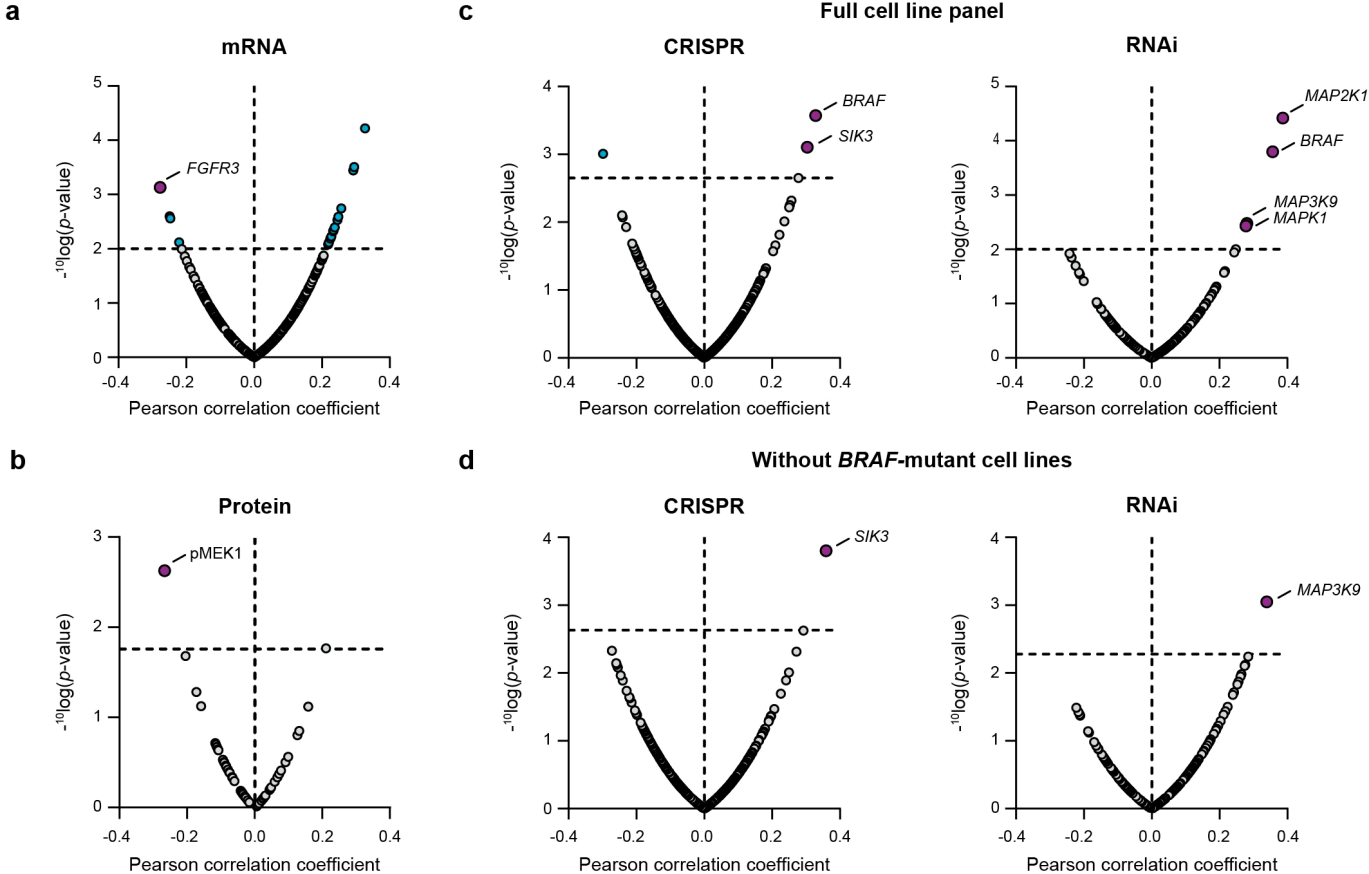
Bioinformatic correlation analyses of cancer cell line sensitivity to nemtabrutinib. **(a)** Volcano plot of Pearson correlation coefficients of IC_50_ values in cell viability assays and basal expression levels of 371 cancer genes. **(b)** Volcano plot of Pearson correlation coefficients of IC_50_ values in cell viability assays and expression levels of 55 cancer proteins. **(c)** Volcano plot of Pearson correlation coefficients of response profiles and gene dependency scores from gene knock-out (CRISPR) or knock-down (RNAi) screens. **(d)** Results from same analysis as in panel c, after exclusion of the 16 *BRAF*-mutant cell lines.

Basal gene expression levels of 371 cancer genes were correlated to the cancer cell line sensitivity of nemtabrutinib. This correlation analysis revealed increased expression of fibroblast growth factor receptor 3 (*FGFR3*) as a significant marker of drug response (**Figure 4a**). Cancer cell line sensitivity was also correlated to the protein expression levels of 55 cancer genes. This showed that high levels of phosphorylated MEK1 are significantly correlated with sensitivity to nemtabrutinib (**Figure 4b**).

Finally, we compared the drug sensitivity profile of nemtabrutinib with the genetic dependency scores of kinase genes available from large-scale CRISPR (465 genes) and RNAi screens (204 genes). Significant positive correlations were found between the IC_50_ profile of nemtabrutinib and the CRISPR knock-out dependency scores of two genes: *BRAF* and *SIK3* (**Figure 4c**). Knock-down of *BRAF* by RNAi was also significantly correlated with response to nemtabrutinib. Additionally, response to nemtabrutinib exhibits a strong positive correlation with knock-down dependency scores of *MAP2K1, MAP3K9*, and *MAPK1* (**Figure 4c**), encoding the MAPK pathway components MEK1, MLK1 and ERK2. The correlation analysis was repeated with the dataset minus the 16 *BRAF-*mutant cell lines to determine whether the preferential targeting of *BRAF-*mutant cell lines by nemtabrutinib may have strongly influenced the results. The significant positive correlations with *SIK3* and *MAP3K9* in the CRISPR and RNAi screen dataset, respectively, were maintained in this analysis (**Figure 4d**), indicating that the dependency of cell lines on these kinases can predict nemtabrutinib response independent of *BRAF* mutation status.

### 3.4 Kinase biochemical inhibition

To determine whether the kinases identified in the bioinformatic analyses of the cell panel profiling study were genuine biochemical targets of nemtabrutinib, kinase enzyme activity assays were performed. The IC_50_ values of nemtabrutinib in biochemical assays with these kinases are given in **Table 1**. **Table 1** summarizes a subset of kinases that were included in the panel of 254 wild-type kinases for the single concentration profiling (**Supplementary Table S3**), such as FGFR1-4, as well as kinases that were not included in this panel (MEK1, MLK1, ERK2, B-RAF and SIK3).

The most sensitive cell line EoL-1 has a constitutively active PDGFRα due to a FIP1L1-PDGFRα fusion (37). In a biochemical assay, PDGFRα was inhibited with an IC_50_ of 12.4 nmol/L. As mentioned before, the gene expression analysis revealed a correlation between high expression of *FGFR3* and cell line sensitivity to nemtabrutinib (**Figure 4a**). Biochemically, nemtabrutinib inhibits FGFR3 with an IC_50_ of 70.1 nmol/L and FGFR2 is even more potently inhibited with an IC_50_ of 34.0 nmol/L. For FGFR1, moderately potent inhibition was found (IC_50_ = 134 nmol/L), while FGFR4 was not potently inhibited (IC_50_ = 1.40 µmol/L). The cell lines AN3-CA and KATO III have an *FGFR2* mutation or amplification, respectively (6). These cell lines were inhibited by nemtabrutinib with a potency within the range of inhibition of the BTK-driven cell lines (IC_50_ = 1.1 µmol/L) (**Supplementary Table S4**). The *FGFR1* fusion-positive cell line KG-1 and *FGFR3* fusion-positive cell line RT-4 (6) are also inhibited within that range, however with a slightly lower potency (IC_50_ = 1.4 and 1.7 µmol/L, respectively). No binding to SIK3 by nemtabrutinib was found (**Table 1**). These results suggest that platelet-derived and fibroblast growth factor receptors are potential drug response biomarkers for nemtabrutinib.

The sensitivity of *BRAF*-mutant cell lines to nemtabrutinib indicates activity of nemtabrutinib on the MAPK pathway. Nemtabrutinib inhibited MEK1 and MEK2 with IC_50_ values of 8.5 and 9.5 nmol/L, respectively (**Table 1**). The compound also showed inhibitory activity in the B-RAF assay with an IC_50_ of 73 nmol/L. It should be noted that the B-RAF kinase assay is a cascade assay, in which activity is indirectly measured via MEK1 and ERK2. Nemtabrutinib did not inhibit MLK1, ERK1 or ERK2 (**Table 1**).

### 3.5 Kinase binding and computational modeling

**Figure 5:**
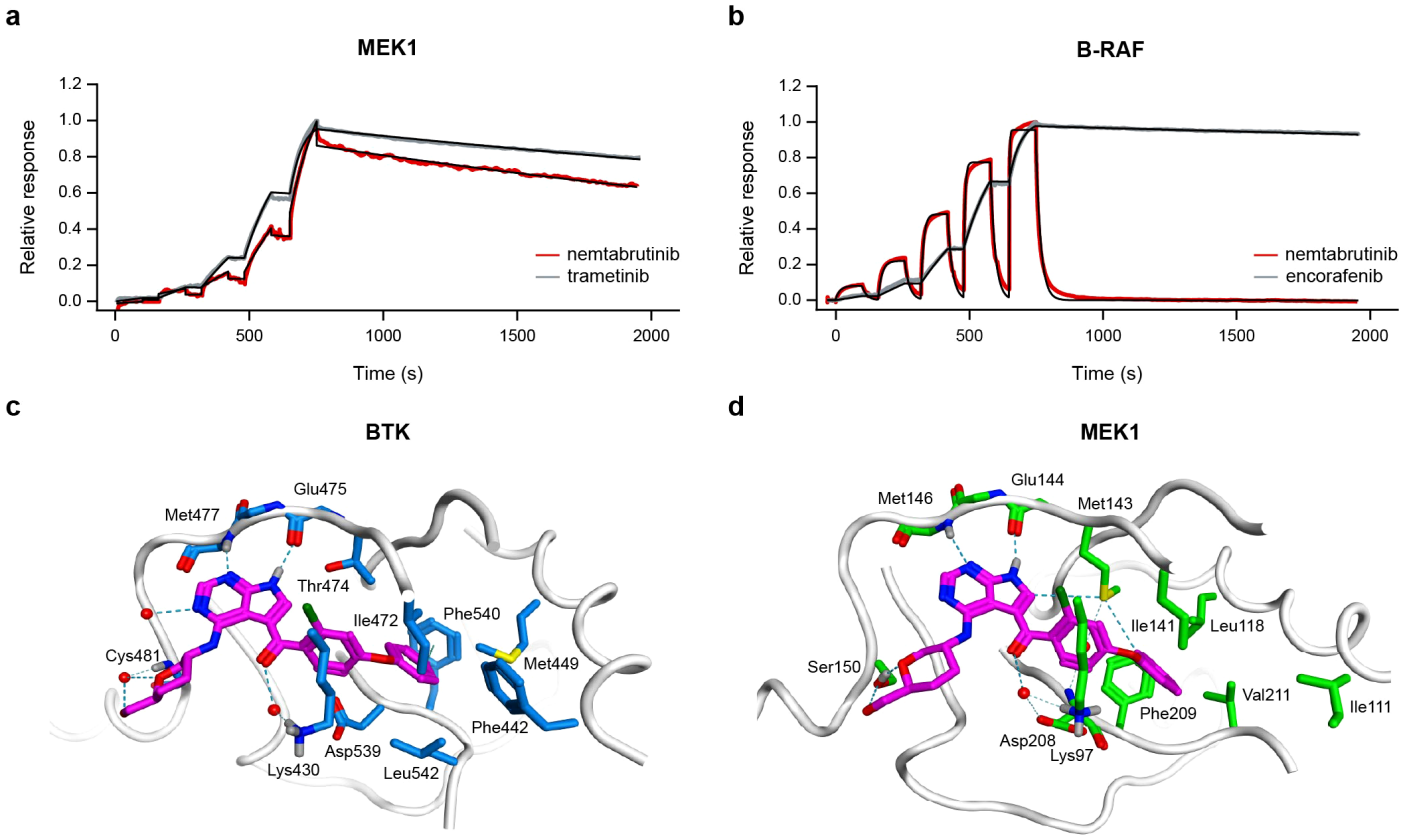
Kinase binding experiments and molecular docking of nemtabrutinib. Sensorgrams of nemtabrutinib and reference inhibitor binding in surface plasmon resonance experiments with MEK1 **(a)** or B-RAF **(b)**. The red and grey lines represent the experimental results and the black lines represent the fits obtained using a 1:1 binding model. **(c)** Binding mode of nemtabrutinib in the ATP-binding pocket of BTK (PDB ID: 6E4F). **(d)** Highest-scoring docking pose of nemtabrutinib in the ATP-binding pocket of MEK1 (PDB ID: 7XLP).

To investigate the specific target of nemtabrutinib in the MAPK pathway, surface plasmon resonance binding experiments were performed on B-RAF and MEK1. Nemtabrutinib bound inactive MEK1 with an affinity (*K*_D_) of 10 nmol/L (**Figure 5a**), whereas it bound activated B-RAF with a *K*_D_ of 1.2 µmol/L (**Figure 5b**), corresponding to a 120-fold difference in affinity (**Supplementary Table S6**). This indicates that the inhibitory effect on MAPK signaling is most likely due to inhibition of MEK1 rather than B-RAF.

X-ray crystallographic studies on nemtabrutinib-bound BTK indicate that nemtabrutinib is an ATP-competitive BTK inhibitor (**Figure 5c**, **Supplementary Table S7**) (15). To investigate the binding mode of nemtabrutinib in MEK1, molecular docking in both the ATP- and allosteric binding pocket of MEK1 was performed. Docking of nemtabrutinib in the ATP-binding pocket of MEK1 resulted in multiple docking poses, which were described by interaction fingerprints (IFPs). These *in silico* predicted IFPs were compared to the IFP derived from the X-ray crystallographic analysis of the nemtabrutinib-BTK complex (15). Interestingly, the highest-scoring docking pose of nemtabrutinib in MEK1 has a very similar IFP as the IFP found in the crystal structure of nemtabrutinib-bound BTK (**Figures 5c, d**, **Supplementary Table S7**). This includes two hydrogen bond interactions between the pyrrolo-pyrimidine ring of nemtabrutinib and two hinge region residues in the ATP-binding pocket of BTK (Met^477^ and Glu^475^) and MEK1 (Met^146^ and Glu^144^). Additionally, the hydroxymethyl-oxane moiety and the carbonyl oxygen atom of nemtabrutinib forms a (water-mediated) hydrogen bond interaction with the residues Cys^481^ and Lys^430^ (BTK) or Ser^150^ and Lys^97^ (MEK1), respectively.

In contrast, docking of nemtabrutinib in the allosteric pocket of MEK1 indicates that nemtabrutinib exhibits extensive sampling of the allosteric pocket, suggesting a lack of strong and highly complementary interactions. Oppositely, the crystal structure of cobimetinib in the allosteric pocket of MEK1 showed multiple polar contacts, such as hydrogen bond interactions with Lys^97^, Asp^190^ and Met^143^ as well as halogen bond interactions with Val^127^ and Ser^212^ (**Supplementary Figure S2A**). Indeed, the IFP analysis shows that nemtabrutinib is unable to form the contacts observed with cobimetinib (**Supplementary Figure S2B, C**). Altogether, the molecular docking experiments suggest that nemtabrutinib preferentially binds to the ATP-binding pocket and does not exhibit strong binding to the allosteric pocket.

## 4 Discussion

Both mutations in kinase genes and their increased expression can cause aberrant tumor growth. To identify the kinases responsible for the anti-proliferative activity of nemtabrutinib in cell lines, we determined associations between cancer cell line sensitivity and gene mutation status. Additionally, we compared the IC_50_ profile of nemtabrutinib with the profiles of other kinase inhibitors profiled on the same panel. Furthermore, we evaluated the correlation of the IC_50_ values with gene expression levels, protein expression levels and CRISPR or RNAi gene dependency scores. The inhibitory activity of nemtabrutinib on the identified candidate kinase targets was investigated in biochemical assays and binding to selected targets was evaluated with surface plasmon resonance and molecular docking studies.

In our study, nemtabrutinib showed moderate cellular activity on the BTK-dependent SU-DHL-6 and REC-1 cell lines. Nemtabrutinib was approximately 40- to 200-fold (SU-DHL-6) or 50- to 4000-fold (REC-1) less potent in cell viability assays with these cell lines than approved BTK inhibitors (**Supplementary Table S5**). It should be noted that BTK inhibitors exert their therapeutic activity by promoting egress of malignant B-cells from lymph nodes (40, 41). Inhibition of tumor cell proliferation is not thought to significantly contribute to their clinical efficacy. Cell viability assays are thus, at best, only a surrogate readout for the activity of BTK inhibitors in B-cell cancers. However, viability assays can be used to identify other therapeutic indications in solid tumors.

Out of the 160 profiled cell lines, nemtabrutinib most potently inhibited the viability of a chronic eosinophilic leukemia cell line (EoL-1), which expresses a chimeric kinase of FIP1L1 and PDGFRα (37) and is, according to the DepMap database (7), strongly dependent on *SIK3*. While the cellular IC_50_ profile of nemtabrutinib correlates with CRISPR dependency of *SIK3*, nemtabrutinib does not bind SIK3 in a biochemical kinase assay. Most likely, the correlation between *SIK3* knock-down dependency and the cell panel profiling results is dominated by the potent inhibitory activity on the EoL-1 cell line (**Supplementary Figure S3A**). In contrast, high *FGFR3* gene expression correlated with sensitivity to nemtabrutinib across the whole cell line panel (**Supplementary Figure S3B**). Cross-reactivity of nemtabrutinib with other growth factor RTKs has been described previously, *i.e.*, with neurotrophic tyrosine receptor kinases (NTRK) (42) and fms-related tyrosine kinase 3 (FLT3) (43). Elgamal et al. explored inhibition of FLT3 by nemtabrutinib as a potential treatment option of acute myeloid leukemia (AML) in preclinical models.

In the first article describing nemtabrutinib, therein referred to as ARQ 531, Reiff et al. reported a number of cross-reactivities with other protein kinases besides BTK. Profiling was performed at an ATP concentration of 1 mmol/L ATP, thus mimicking intracellular ATP levels (44). An IC_50_ of 599 nmol/L was determined in an enzyme assay for MEK1. We used the same protein and assay readout (both from Carna) but performed the assay at *K*_M,bin_, *i.e.*, an ATP concentration close to *K*_M,ATP_, which for MEK1 and MEK2 corresponded to 10 µmol/L ATP. Although the cellular ATP concentration is generally between 1–5 mmol/L, using the *K*_M,ATP_ in biochemical assays allows the IC_50_ value to become a direct measure of the binding affinity between the investigated compound and the kinase (45). We determined an IC_50_ of approximately 9 nmol/L for MEK1 and MEK2, which is six times higher than its IC_50_ in the enzyme assay on BTK (**Table 1**). Reiff et al. reported that nemtabrutinib inhibited MEK1 with a 1200 times higher IC_50_ than BTK. The cross-reactivity of nemtabrutinib with MEK1 and MEK2 corresponds with the preferential targeting of *BRAF*-mutant cell lines. Of note, we independently confirmed the higher IC_50_ on MEK1 in the biochemical enzyme assay at 1 mmol/L ATP (*i.e.*, 456 nmol/L; **Table 1**).

As mentioned before, X-ray crystallographic studies of nemtabrutinib with BTK indicate that nemtabrutinib is an ATP-competitive (Type I) inhibitor (15). Our docking studies indicate that it binds in a similar way to MEK1. In contrast, currently approved MEK inhibitors, such as trametinib and cobimetinib, target MEK through an allosteric pocket adjacent to the ATP-binding pocket (Type III inhibitors) (46). Allosteric MEK1 inhibitors are used in combination with B-RAF inhibitors for treating *BRAF* V600-mutant cancers or as monotherapies for neurofibromatosis type I (47). However, resistance to allosteric MEK inhibitors is frequently observed and can arise through multiple mechanisms. First, mutations in MEK1 or MEK2 can confer cross-resistance to multiple inhibitors within this class. Notably, many of these resistance mutations remain susceptible to ATP-competitive, Type I MEK inhibitors (48, 49). Second, allosteric MEK inhibitors exhibit reduced binding affinity for phosphorylated MEK, which can be caused by overexpression of *BRAF* (35, 50). In contrast, ATP-competitive inhibitors retain their affinity against phosphorylated MEK, underscoring another advantage of this binding mode (35).

In addition to the ATP-competitive binding mode of nemtabrutinib, its multi-kinase inhibition profile may provide therapeutic benefits beyond those of selective MEK inhibitors. Reactivation of the MAPK pathway, commonly driven by diminished ERK-mediated negative feedback on RTKs or acquired RTK overexpression, is a prevalent resistance mechanism (51, 52). Our findings indicate that nemtabrutinib not only targets MEK but also inhibits multiple RTKs. This dual mechanism of action can potentially delay MAPK pathway reactivation and extend the duration of response compared to more selective allosteric or ATP-competitive MEK inhibitors.

Taken together, our results show that nemtabrutinib downregulates MAPK signaling by inhibiting MEK1 and that it is also an inhibitor of several growth factor RTKs, including FGFR2, FGFR3 and PDGFRα. The ATP-competitive binding mode of nemtabrutinib in MEK and its dual mechanism of action are promising features that warrant further investigation in MAPK-driven cancers. Our data illustrate the power of combining cell panel profiling, bioinformatics and kinase biochemical profiling for the identification of differentiators and new potential therapeutic applications of kinase inhibitors.

## Data Availability

The original contributions presented in the study are included in the article/**Supplementary Material**. Further inquiries can be directed to the corresponding author.
