# Supplementary figures and images for: Combined cellular and biochemical profiling of Bruton’s tyrosine kinase inhibitor nemtabrutinib reveals potential application in MAPK-driven cancers
Source: Front Oncol. 2025 Oct 22;15:1667291. doi: 10.3389/fonc.2025.1667291 (PMC12586182; doi:10.3389/fonc.2025.1667291)

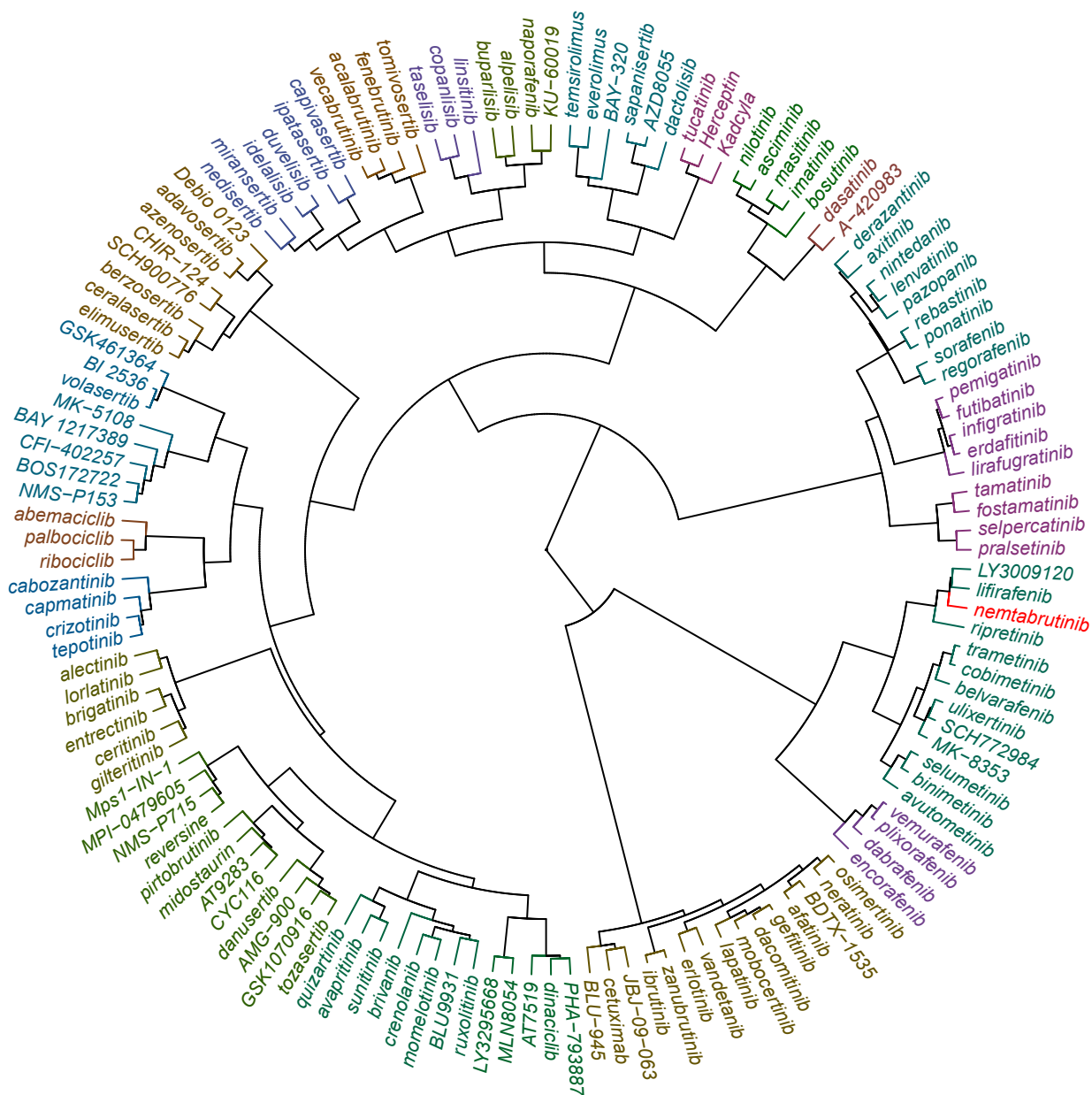

Supplement: Supplementary file 1 [file DataSheet1.pdf]
