# Supplementary material for: Combined cellular and biochemical profiling of Bruton’s tyrosine kinase inhibitor nemtabrutinib reveals potential application in MAPK-driven cancers
Source: Front Oncol. 2025 Oct 22;15:1667291. doi: 10.3389/fonc.2025.1667291 (PMC12586182; doi:10.3389/fonc.2025.1667291)
Supplement: Supplementary file 2 [file DataSheet2.pdf]

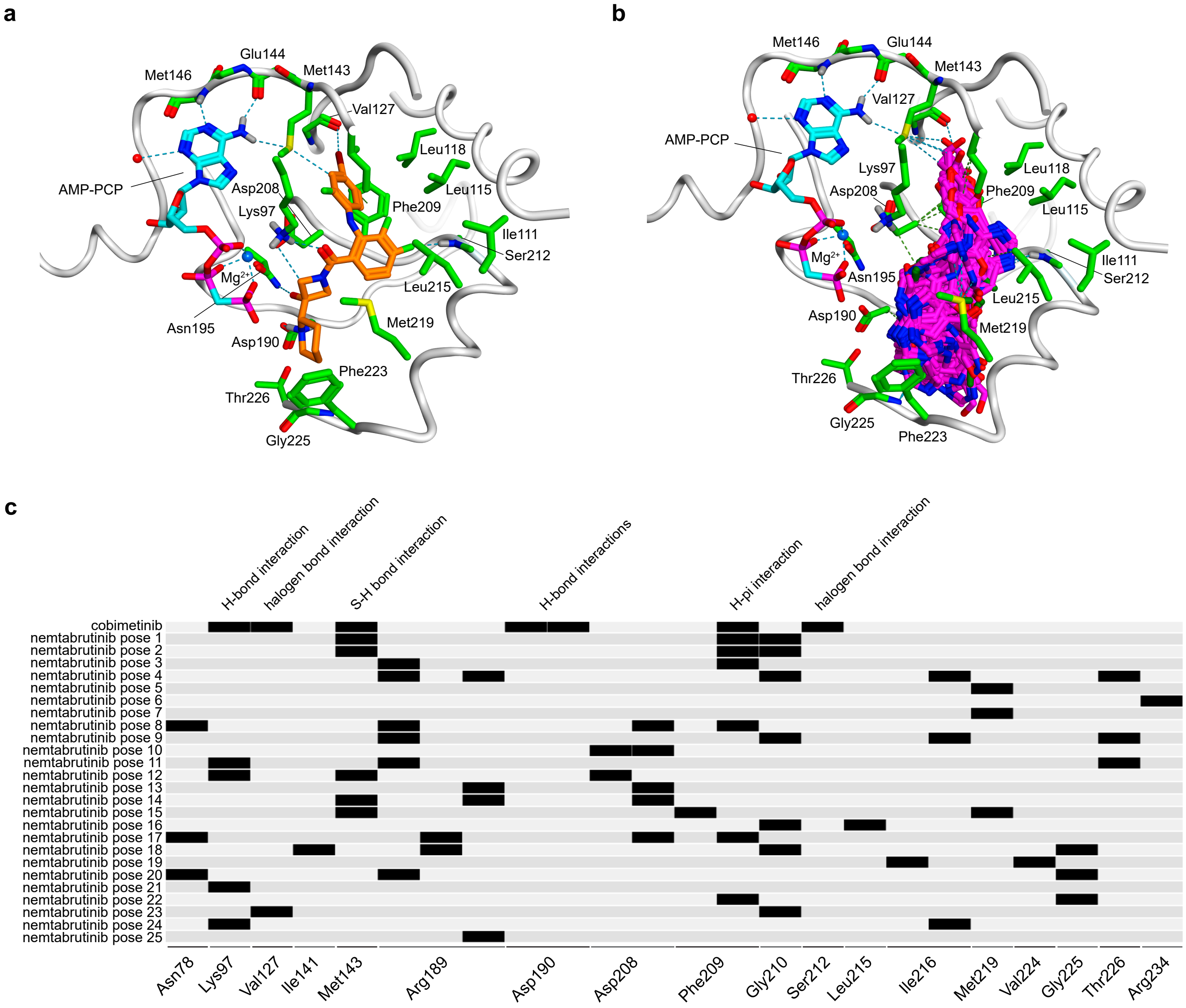

Supplementary Figure S2. Docking of nemtabrutinib in the allosteric binding site of MEK1. a. Binding mode of the allosteric MEK1 inhibitor cobimetinib in MEK1 (PDB ID: 4AN2). **b.** Overlay of the 25 highest-scoring docking poses of nemtabrutinib in the allosteric binding site of MEK1 (PDB ID: 4AN2). **c.** Interaction fingerprint of cobimetinib and the 25 docking poses of nemtabrutinib in the allosteric binding site of MEK1.
