# Supplementary material for: Combined cellular and biochemical profiling of Bruton’s tyrosine kinase inhibitor nemtabrutinib reveals potential application in MAPK-driven cancers
Source: Front Oncol. 2025 Oct 22;15:1667291. doi: 10.3389/fonc.2025.1667291 (PMC12586182; doi:10.3389/fonc.2025.1667291)
Supplement: Supplementary file 3 [file DataSheet3.pdf]

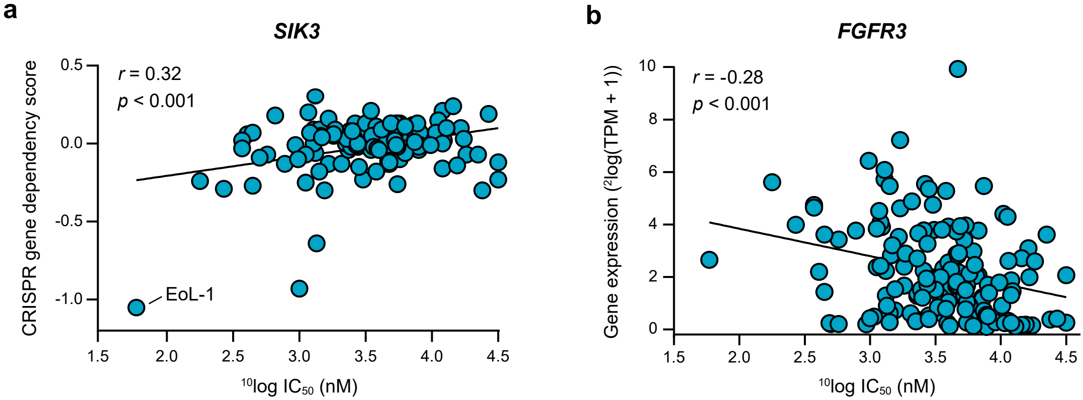

Supplementary Figure S3. a. Correlation between gene dependency scores of *SIK3* and IC_{50} values of nemtabrutinib. **b.** Correlation between gene expression levels of *FGFR3* and IC_{50} values of nemtabrutinib.
