# Supplementary material for: Combined cellular and biochemical profiling of Bruton’s tyrosine kinase inhibitor nemtabrutinib reveals potential application in MAPK-driven cancers
Source: Front Oncol. 2025 Oct 22;15:1667291. doi: 10.3389/fonc.2025.1667291 (PMC12586182; doi:10.3389/fonc.2025.1667291)
Supplement: Supplementary file 8 [file Table5.pdf]

Supplementary Table S5: IC_{50} values and IC_{50} fold changes of nemtabrutinib with the four approved BTK inhibitors on the BTK-driven cell lines SU-DHL-6 and REC-1.

**Inhibitor**	**SU-DHL-6**		**REC-1**	
	IC_{50} (nmol/L)	fold change	IC_{50} (nmol/L)	fold change
acalabrutinib	3.1	186	21	96
ibrutinib	12	48	0.51	4001
zanubrutinib	3.8	153	3.1	657
nemtabrutinib	577	-	2041	-
pirtobrutinib	13	43	38	53
