# Supplementary material for: Combined cellular and biochemical profiling of Bruton’s tyrosine kinase inhibitor nemtabrutinib reveals potential application in MAPK-driven cancers
Source: Front Oncol. 2025 Oct 22;15:1667291. doi: 10.3389/fonc.2025.1667291 (PMC12586182; doi:10.3389/fonc.2025.1667291)
Supplement: Supplementary file 9 [file Table6.pdf]

Supplementary Table S6: Surface plasmon resonance binding kinetics of nemtabrutinib and encorafenib on B-RAF, and nemtabrutinib and trametinib on MEK1.

Kinase	Inhibitor	k_a (L/mol*s)	log(k_a)	SD log(k_a)	k_d (1/s)	log (k_d)	SD log (k_d)	K_D (mol/L)	$t_{1/2}$ (s)	No. of exp.
B-RAF	nemtabrutinib	4.58×10^{4}	4.66	0.002	5.54×10^{-2}	-1.26	0.00002	1.21×10^{-6}	12.5	2
	encorafenib	2.27×10^{5}	5.36	0.04	4.00×10^{-5}	-4.40	0.20	1.76×10^{-10}	17300	3
MEK1	nemtabrutinib	3.18×10^{4}	4.50	0.39	3.23×10^{-4}	-3.49	0.12	1.02×10^{-8}	2140	5
	trametinib	2.20×10^{5}	5.34	0.02	1.35×10^{-4}	-3.87	0.09	6.13×10^{-10}	5140	2

k_a = Association rate
k_d = Dissociation rate
K_D = Affinity
No. of exp. = Number of experiments
SD = Standard deviation
$t_{1/2}$ = Half-life
