# Supplementary material for: Combined cellular and biochemical profiling of Bruton’s tyrosine kinase inhibitor nemtabrutinib reveals potential application in MAPK-driven cancers
Source: Front Oncol. 2025 Oct 22;15:1667291. doi: 10.3389/fonc.2025.1667291 (PMC12586182; doi:10.3389/fonc.2025.1667291)
Supplement: Supplementary file 10 [file Table7.pdf]

Supplementary Table S7: Interactions of nemtabrutinib in the ATP-binding pocket of the crystal structure of BTK and highest-scoring docking pose of MEK1.

Protein	Amino acid	Interaction type
BTK	Lys430	H_2O mediated hydrogen bond
	Glu475 (hinge residue)	Hydrogen bond
	Met477 (hinge residue)	Hydrogen bond
	Cys481	H_2O mediated hydrogen bond
	Phe540	H-pi interaction
MEK1	Lys97 / Asp208	H_2O mediated hydrogen bond
	Met143 (gatekeeper)	S-H bond
	Glu144 (hinge residue)	Hydrogen bond
	Met146 (hinge residue)	Hydrogen bond
	Ser150	Hydrogen bond
